# Hybrid healthcare unit recommendation system using computational techniques with lung cancer segmentation

**DOI:** 10.3389/fmed.2024.1429291

**Published:** 2024-07-19

**Authors:** Eid Albalawi, Eali Stephen Neal Joshua, N. M. Joys, Surbhi Bhatia Khan, Hadil Shaiba, Sultan Ahmad, Jabeen Nazeer

**Affiliations:** ^1^Department of Computer science, College of Computer Science and Information Technology, King Faisal University, Hofuf, Saudi Arabia; ^2^Department of Computer Science & Engineering, Gandhi Institute of Technology and Management (GITAM) (Deemed to be University), Visakhapatnam, Andhra Pradesh, India; ^3^Department of Computer Science & Engineering, Anil Neerukonda Institute of Technology and Sciences, Visakhapatnam, Andhra Pradesh, India; ^4^School of Science, Engineering and Environment, University of Salford, Manchester, United Kingdom; ^5^Department of Electrical and Computer Engineering, Lebanese American University, Byblos, Lebanon; ^6^Department of Computer Science, College of Computer and Information Sciences, Princess Nourah Bint Abdulrahman University, Riyadh, Saudi Arabia; ^7^Department of Computer Science, College of Computer Engineering and Sciences, Prince Sattam Bin Abdulaziz University, Alkharj, Saudi Arabia; ^8^School of Computer Science and Engineering, Lovely Professional University, Phagwara, Punjab, India

**Keywords:** image segmentation, two-parameter logistic type distribution, performance evaluation, CLAHE, ROI segmentation, lung cancer detection

## Abstract

**Introduction:**

Our research addresses the critical need for accurate segmentation in medical healthcare applications, particularly in lung nodule detection using Computed Tomography (CT). Our investigation focuses on determining the particle composition of lung nodules, a vital aspect of diagnosis and treatment planning.

**Methods:**

Our model was trained and evaluated using several deep learning classifiers on the LUNA-16 dataset, achieving superior performance in terms of the Probabilistic Rand Index (PRI), Variation of Information (VOI), Region of Interest (ROI), Dice Coecient, and Global Consistency Error (GCE).

**Results:**

The evaluation demonstrated a high accuracy of 91.76% for parameter estimation, confirming the effectiveness of the proposed approach.

**Discussion:**

Our investigation focuses on determining the particle composition of lung nodules, a vital aspect of diagnosis and treatment planning. We proposed a novel segmentation model to identify lung disease from CT scans to achieve this. We proposed a learning architecture that combines U-Net with a Two-parameter logistic distribution for accurate image segmentation; this hybrid model is called U-Net++, leveraging Contrast Limited Adaptive Histogram Equalization (CLAHE) on a 5,000 set of CT scan images.

## 1 Introduction

Lung cancer begins in the lungs and spreads throughout the rest of the body (1), including the brain. Lung cancer is the most common cause of cancer-related mortality worldwide (2). Although lung cancer is more frequent in smokers, it may also occur in nonsmokers (3). The incidence of lung cancer is often and excessively increased with smoking. Lung cancer risk may be lowered even if you have smoked for a long period. Segmentation, a type of image compression, is necessary to infer information from photos. Imaging modalities (4), including Magnetic Resonance Imaging (MRI) and Computed Tomography (CT), can be utilized to create Computer-Aided Diagnostic (CAD) (5) models that can be used to diagnose and treat patients in precision medicine. Using a limited quantity of medical image data, we demonstrated the efficacy of our proposed model, which we refer to as U-NET++. A method known as the dice coefficient loss was used to compute the findings of the investigations. An approach to labeling preprocessing that is in line with the approaches that are already in use is presented in this paper.

The main novelty of this study is as follows.

- To propose the segmentation model for identifying lung disease made on CT scans with the limited set of CT scan images using the CLAHE.
- To develop the learning architecture combining U-Net with a two-parameter logistic distribution for image segmentation, was used for segmentation.
- To train the models using several deep learning classifiers and evaluate the performance of the models using benchmarks on the LUNA16 dataset using different information retrieval metrics.

The following section describes the organization of the subsequent sections of this study.

A considerable amount of important research is presented in Section 2. Deep learning architectures are used in segmenting medical images by U-NET++, which is created by combining the two-parameter model recommended with distribution learning of the U-Net type. Section 3 provides a comprehensive explanation of the topic. At this point, the criteria for evaluating the model's performance discussed in the fourth part of the section are presented.

## 2 Related works

A meta-analysis of the literature was performed. Table 1 clearly shows the literature matrix representation of their meta-analysis and the strong relationships between the authors and their respective works. CT scans were assessed based on the image brightness. Different areas of the same region should have the same intensity; hence, segmentation is an effective method to separate objects. Various segmentation procedures were found to be useful in this study. Three-step segmentation-based strategy for distinguishing lung regions.

**Table 1:** Presents the related study and limitations in the works.

**References**	**Dataset**	**Split**	**Key arguments**	**Drawbacks**
Huang and Hu (6)	Lung Nodule Analysis 2016 dataset and Alibaba Tianchi Lung Cancer Detection Competition dataset.	60:40	The Noisy U-Net (NU-Net) increases the diagnosis of early lung cancer nodules by increasing the sensitivity to tiny nodules measuring between 3 and 5 mm in diameter. This is achieved by adding distinct noise to hidden layers during training.	Insufficient validation across a number of clinical situations or datasets has been done to evaluate NU-Net's applicability and robustness. The practical application is restricted since it ignores false positives and the algorithm's inconsistent performance with diverse nodule properties. The lack of advanced method comparison studies limits NU-Net's effectiveness compared to U-Net.
Zhao et al. (7)	LUNA-16	70:30	The proposed approach for accurately detecting cancerous lung lesions from CT scans involves using a patch-based 3D U-Net and a contextual convolutional neural network.	The article lacks a thorough validation or explanation of the model's performance variability across various datasets or in real-world clinical situations. Furthermore, the lack of a comparison to current approaches hinders the ability to assess the superiority or applicability of the proposed strategy.
Chiu et al. (8)	LUNA-16	70:30	The 2D U-Net approach effectively identifies lung nodules in medical pictures. The detection performance may be improved by utilizing ROI segmentation models and further labeling.	The use of the ROI segmentation technique enhances the accuracy of lung nodule identification. The U-Net-based network architecture demonstrates high proficiency in segmenting lung nodules. Additionally, complementary labeling appears to be helpful in situations when there is a scarcity of data.
Gao et al. (9)	LUNA-16	70:30	The U-Net model, which incorporates an attention mechanism and residual structure, effectively segments lung cancer bone metastases in SPECT images, improving early identification and treatment outcomes.	The research will likely neglect practical factors, such as variations in SPECT imaging circumstances or anomalies that may undermine the model's robustness in real clinical settings.
Cai et al. (10)	LUNA-16	60:40	The U-Net deep learning network consistently enables the identification of lung cancer nodules larger than 3 mm in diameter, hence facilitating the progress of early detection and therapy methods for this disease.	The research work fails to describe the AI model's clinical validation and integration in real-world healthcare settings, obscuring its practicality. It prioritizes model accuracy above false positives and negatives, which are essential for successful practical diagnosis. Due to its architecture and lack of testing against more adaptable modern methods, the U-Net and PSP Net AI models' effectiveness is unknown. Due to its dataset dependence, the model may not work for all patient groups or imaging situations (Luna16).
Banu et al. (11)	LUNA-16	70:30	The use of WEU-Net, also known as weight excitation U-Net, enhances the early identification of lung cancer by precisely segmenting lung nodules in CT images.	The work does not explain how the model shows nodule variety, size, and consistency across datasets. The therapeutic adoption of this technology depends on time efficiency and computational needs, which are being disregarded. The lack of a comparison with other cutting-edge segmentation methods hinders our comprehension of WEU-Net's efficacy. To conclude, the model's interpretability and therapeutic potential in diagnostic and treatment planning are undisputed.
Xia (12)	LUNA-16	60:40	When it comes to detecting supplemental lung cancer, RUNet image segmentation outperforms 3D U-Net. Pro-CRP, CEA, and NSE serve as diagnostic markers for malignant lung cancers.	The research lacks a thorough examination of any biases or confounding variables that may impact the accuracy of diagnoses and the performance of the model when selecting patients. The research did not assess the generalizability of the findings to larger groups of patients or other imaging techniques other than MRI. The absence of a comparative analysis with other verified segmentation approaches impedes the understanding of the specific benefits that RUNet offers in contrast to other methods. Moreover, there is insufficient information about using the model in clinical settings to verify its effectiveness in real-world situations or with external datasets.
Chhabra et al. (13)	IIITD-CLF	8:2	The study discusses how regularization and patch size affect how well the model works. segmentation with different network designs and patch sizes to make it more accurate.	Factors like scalability, external validity, possible bias, and limited generalizability should be considered.
Venkatesh et al. (14)	LIDC-IDRI	70:30	It aims to revolutionize the detection of lung cancer by offering a more accurate and efficient approach compared to existing approaches.	The evaluation of the effectiveness of the suggested technique in relation to existing methods is limited due to the lack of a comparative study with state-of-the-art systems. Further investigation is required to enable the idea's implementation in real-world clinical environments, considering ethical concerns, regulatory challenges, and the potential to scale up.
Madhu et al. (15)	POCUS	70:30	This paper presents XCovNet, an improved Xception neural network, which outperforms existing deep learning models for point-of-care lung ultrasound data analysis, enabling accurate identification of COVID-19.	The study enhances medical imaging technology for the detection of infectious diseases by developing XCovNet and showcasing its improved performance in comparison to current models. This is essential to fulfill the need for accurate and expedient diagnostic tools in contexts with limited resources.
Lamba et al. (16)	GSCE25066	70:30	The aim of the project is to use machine learning techniques to find crucial genes for cancer subtyping. These genes will then be validated using the Kaplan-Meier Survival Model.	The study paper does not explicitly discuss any recognized research constraints in the categorization of breast cancer subtypes based on gene expression data. Subsequent studies in this domain might examine the impact of different feature selection methods on the effectiveness of models and the reliability of findings across different datasets.

First, the lung was segmented using gray-level thresholding. Dynamic programming then divides the lung lobes. Finally, morphology-based smoothing approaches were employed. Region-based segmentation includes enlarging, dividing, and combining the areas (17).

A novel convolutional network type known as U-NET++ was developed to analyze CT images used in the biological sciences. U-NET++ was used in this study to extract lung fields from CT images. In healthcare, U-NET++ is nothing more than a variation of ConvNet, combined with various *ad hoc* data augmentation methods.

The robustness of the model was compromised because the authors of (6–8) carried out their research using the same data potential. The traditional U-Net network (9–16) is a semantic segmentation network built using a fully convolutional neural network. Although it has a relatively small number of layers, the network is nevertheless capable of functioning well, although less complex than its predecessors. The UNET network consists of two main components: down-sampling and up-sampling algorithms. The process of feature extraction, also known as down sampling, involves using convolutional, and pooling layers. This stage is accountable for obtaining characteristics from the original image. A deconvolution technique is employed to enhance the feature map's intricacy. The alternative term for the structure that involves down-sampling and up-sampling is the decoder-encoder structure. The original picture undergoes convolutional and pooling layers during the down-sampling process. This leads to the generation of feature maps that include different levels of information. Regarding visual characteristics, the feature maps exhibit diverse abstraction levels. Combining the down-sampled feature map makes it possible to retrieve a larger portion of the abstract detail information lost during training. As a consequence, the network becomes more successful at segmentation. During the up-sampling process, the deconvolution layer systematically increases the feature image's dimensions. Consequently, the lung's three-dimensional nature results in a substantial loss of spatial information. Consequently, a substantial quantity of relevant information is lost when down-sampling occurs. As retrieving all data is impractical, up-sampling yields imprecise outcomes and disregards visual nuances. Moreover, in addition to the aforementioned concerns, implementing a deep neural network is necessary for future advancement. According to the results of applying U-NET++ to a new dataset, the precision of the IOU and Dice coefficients improved. The test results demonstrate that the U-NET++ architecture improves the efficiency of multiscale conversion and fully connected systems. The authors in (18) propose a novel approach for lung CT scan classification. They combined handcrafted features were extracted using Q-deformed entropy (QDE), which captured image texture based on intensity variations, with features automatically learned by a Convolutional Neural Network (CNN). This fusion strategy aimed to improve the identification of healthy lungs from those affected by conditions like COVID-19 or pneumonia (18). This proposed approach demonstrated the benefits of combining handcrafted and automatically learned features. Segmentation focused the model on relevant lung regions, and the LSTM network effectively utilized the fused features for accurate classification.

## 3 Materials and methods

### 3.1 U-NET++ architectural design

**Figure 1:**
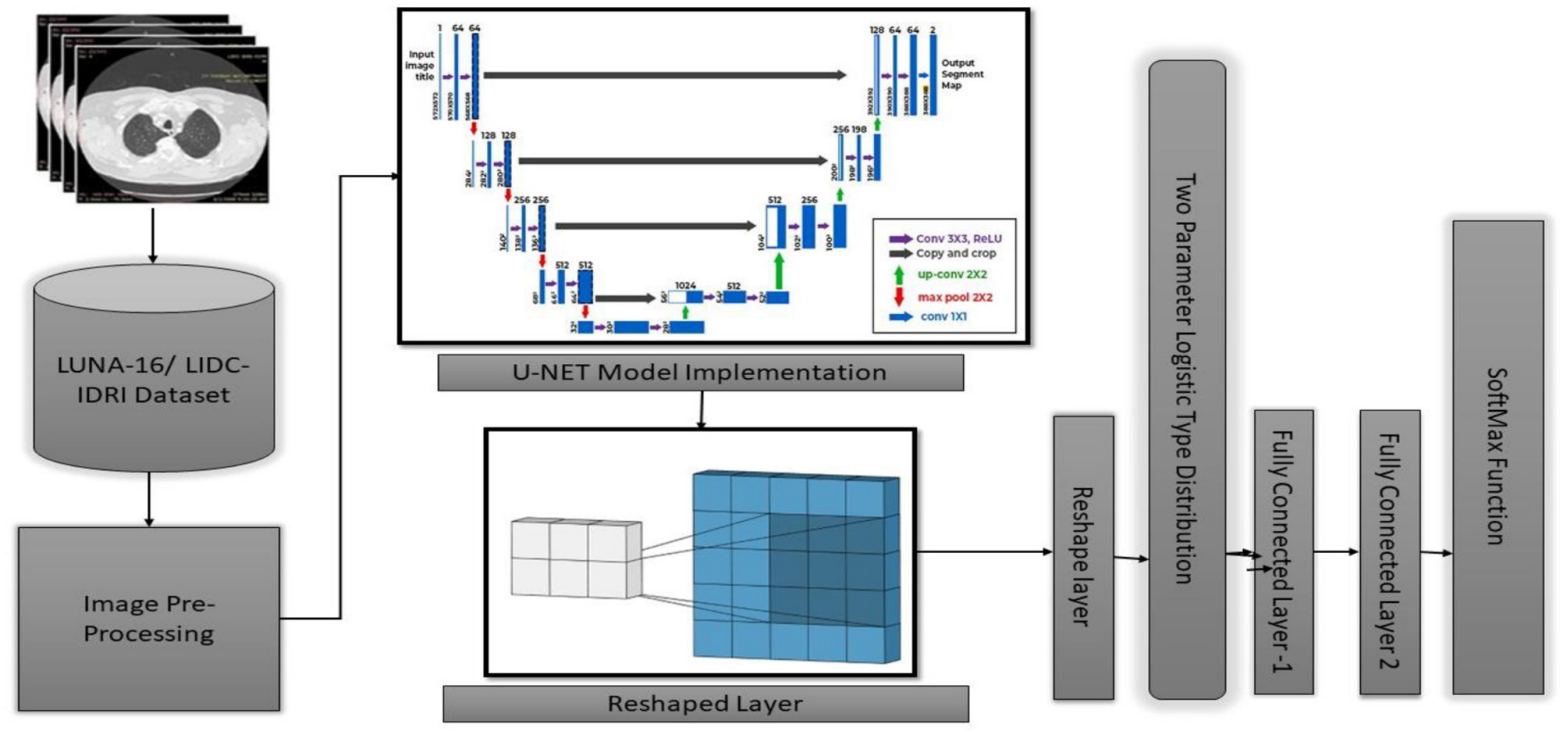
Architecture of U-NET++.

This study introduces the U-NET++ hybrid model, which utilizes a two-parameter logistic function to identify lung nodules from CT scans accurately. Lung CT scans were classified as “benign” or “malignant” when used as an input for a binary classification system. A unique hybrid model that combines U-Net (19) and two-parameter logistic distribution was developed to segment and diagnose lung cancer. The model was generated using the dataset of LUNA-16 lung CT images. The U-NET++ model is highly esteemed as a leading architecture in computer vision, primarily because it is built on established computer vision approaches. When assessed using the ImageNet test dataset, this model achieved a precision rate of 91%. The main architectural improvement in the model is the filter size, an improved version of the U-NET. Figure 1 illustrates the architecture of the proposed model.

**Figure 2:**
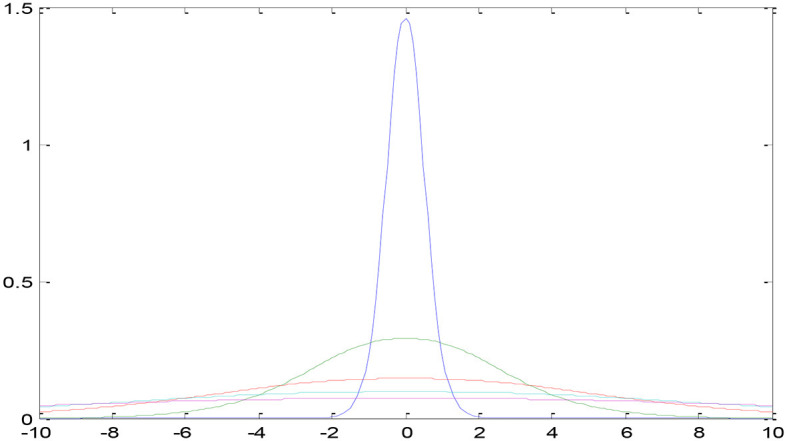
Two-parameter U-NET++ two-parameter type distribution.

In the current section, a detailed presentation of the combination of two- and three-parameter logistic distribution models is presented. Figure 2 shows a two-parameter U-NET++ logistic-type distribution. In general, the pixel intensities are the content through which the quantification of the image details performed on several regions of the images. The brightness of a picture or image can be measured by using several performance metrics such as the moisture in the surroundings, lightening of the images, vision, and the surrounding environmental conditions. This measurement can be performed using the pixel values and pixel intensities. For instance, pixel (*a, b*) intensity measurement was performed using the function *z* = *f* (*a, b*) and considered as a random variable. To better analyze and understand the performance of the currently considered model and the intensities of pixels for various images, the model was designed for both parametric and parametric models. The pdf of the pixel intensity is given by


(1) $$\begin{array}{c} f\left(y,Մ,\Omega^{2}\right)=\frac{\left[\frac{3}{\left(12+\pi^{2}\right)}\right]\left[4+{\left(\frac{y-Մ}{\Omega }\right)}^{2}\right]e^{-\left(\frac{y-Մ}{\Omega }\right)}}{k\left[1+e^{-{\left(\frac{y-Մ}{\Omega }\right)}^{2}}\right]},\\ -\infty <y<\infty ,-\infty <Մ<\infty ,\Omega >0 \end{array}$$

Where *y* is the pixel Intensity, Մ is the mean of pixels s, and omegas the variance of the ented image's pixels.

### 3.2 U-NET++ algorithm

#### 3.2.1 U-Net ith two parameter type distribution $\Omega_{i}{\;}^{2}$

For updating ${\Omega_{i}}^{2}$ we differentiate R(*Q, Q*) with respect to ${\Omega_{i}}^{2}$ and equate it to zero. That is $\frac{\partial }{\partial \Omega^{2}}\left(Q\left(Q,Q^{\left(l\right)}\right)\right)=0$. This implies $E\left[\frac{\partial }{\partial \Omega }\left(\log L\left(Q,Q^{\left(l\right)}\right)\right)\right]=0.$ The derivative was applied and implemented for both parameter models with ${\sigma_{i}}^{2}$ for the two-parameter model, with estimation error of 0.001 and it was with the biased estimation. From the Equations 1–6 segmentation algorithm used in the proposed algorithm.


(2) $$\begin{array}{c} \frac{\partial }{\partial \Omega_{i}{\;}^{2}}\\ \left[\sum_{s=1}^{N}\sum_{i=1}^{K}P_{i}(y_{s.},Q^{l})\log\beta_{i}\frac{\left[\frac{3}{12+\pi^{2}}\right]\left[4+{\left(\frac{y_{s}-{Մ}_{i}}{\Omega_{i}}\right)}^{2}\right]e^{-\left(\frac{y_{s}-{Մ}_{i}}{\Omega_{i}}\right)}}{\Omega_{i}\left[1+e^{-{\left(\frac{y_{s}-{Մ}_{i}}{\Omega_{i}}\right)}^{2}}\right]}\right]=0. \end{array}$$

The updated equations of ${\sigma_{i}}^{2}$ at (*l*+1)^*th*^ iteration is


(3) $$\begin{array}{c} \Omega_{i}{\;}^{2^{\left(l+1\right)}}=\\ \frac{\sum_{s=1}^{N}\left[\left[\frac{\left(y_{s}-{Մ}_{i}{\;}^{\left(l+1\right)}\right)}{\Omega_{i}{\;}^{3^{\left(l\right)}}}\right]-\left[\frac{1}{\Omega_{i}{\;}^{2^{\left(l\right)}}}\right]-\left[\frac{{\left(y_{s}-{Մ}_{i}{\;}^{\left(l+1\right)}\right)}^{2}}{\Omega_{i}{\;}^{4}\left(1+e^{{\left(\frac{y_{s}-{Մ}_{i}{\;}^{\left(l+1\right)}}{\sigma_{i}{\;}^{\left(l\right)}}\right)}^{2}}\right)}\right]\right]p_{i}(y_{s},Q^{\left(l\right)})}{\sum_{s=1}^{N}\frac{\left(x_{s}-\mu_{i}{\;}^{\left(l+l\right)})p_{i}(x_{s},\theta^{\left(l\right)}\right)}{\sigma_{i}{\;}^{4^{\left(l\right)}}(4\sigma_{i}{\;}^{2^{\left(l\right)}}+{\left(x_{s}-\mu_{i}{\;}^{\left(l+1\right)}\right)}^{2})}}. \end{array}$$

For three-parameter logistic type distribution: -


(4) $$\begin{array}{c} \frac{\partial }{\partial \Omega_{i}{\;}^{2}}\\ \left[\sum_{s=1}^{N}\sum_{i=1}^{K}P_{i}(y_{s.},Q^{l})\log\beta_{i}\frac{\left[\frac{3}{3p+\pi^{2}}\right]\left[p+{\left(\frac{y_{s}-{Մ}_{i}}{\Omega_{i}}\right)}^{2}\right]e^{-\left(\frac{y_{s}-{Մ}_{i}}{\Omega_{i}}\right)}}{\Omega_{i}{\left[1+e^{-\left(\frac{y_{s}-{Մ}_{i}}{\Omega_{i}}\right)}\right]}^{2}}\right]=0. \end{array}$$

(5) $$\begin{array}{c} {ϒ}_{i}^{2^{\left(l+1\right)}}=\\ \frac{\sum_{s=1}^{N}\frac{P_{i}(y_{s.},Q^{\left(l\right)})\left(y_{s}-{Մ}_{i}{\;}^{\left(l+1\right)}\right)}{2\Omega_{i}{\;}^{3^{\left(l\right)}}}-\sum_{s=1}^{N}\frac{P_{i}(y_{s.},Q^{\left(l\right)})\left(y_{s}-{Մ}_{i}{\;}^{\left(l+1\right)}\right)}{\sigma_{i}{\;}^{3^{\left(l\right)}}\left[1+e^{\frac{\left(y_{s}-{Մ}_{i}\right)}{\Omega_{i}}}\right]}-\sum_{s=1}^{N}\frac{P_{i}(y_{s.},Q^{\left(l\right)})}{2\Omega_{i}{\;}^{2^{\left(l\right)}}}}{\sum_{s=1}^{N}\frac{P_{i}(y_{s.},Q^{\left(l\right)}){\left(y_{s}-{Մ}_{i}{\;}^{\left(l+1\right)}\right)}^{2}}{\sigma_{i}{\;}^{4^{\left(l\right)}}\left[p\Omega_{i}{\;}^{2^{\left(l\right)}}+{\left(y_{s}-{Մ}_{i}{\;}^{\left(l+1\right)}\right)}^{2}\right]}}. \end{array}$$

Were


(6) $$\begin{array}{l} p_{i}\left(y_{s},Q^{\left(l\right)}\right)=\left[\frac{{\beta_{i}}^{\left(l+1\right)}f_{i}\left(y_{s,}{{ϒ}_{i}}^{\left(l+1\right)},{\Omega_{i}}^{2^{\left(l\right)}}\right)}{\overset{k}{\underset{i=1}{\sum }}{\beta_{i}}^{\left(l+1\right)}f_{i}\left(y_{s},{{ϒ}_{i}}^{\left(l+1\right)},{\Omega_{i}}^{\left(l\right)}\right)}\right]. \end{array}$$

### 3.3 Module design

**Figure 3:**
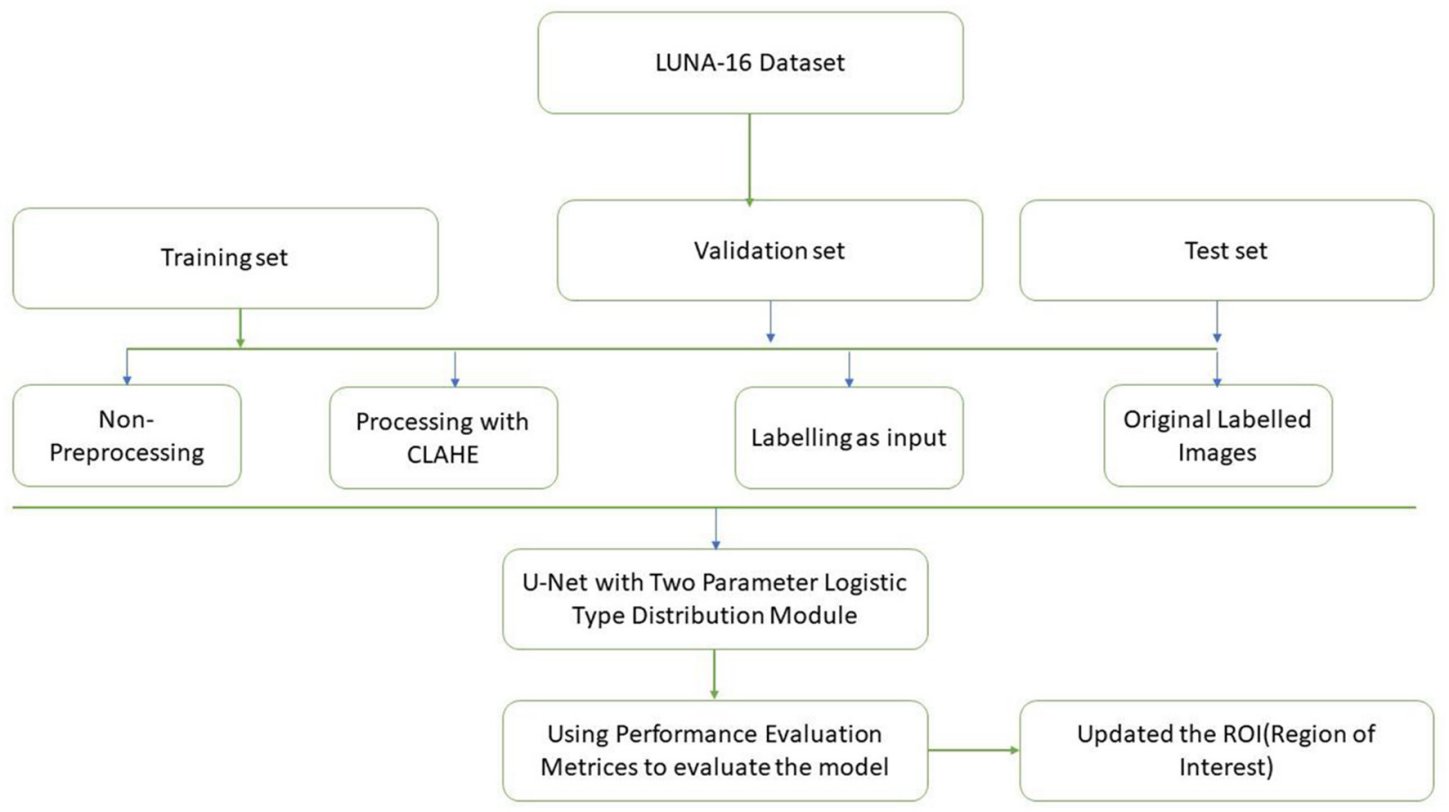
Methodology design.

Figure 3 discuss about the methodology design followed in our proposed work. A typical image processing method is contrast-limited adaptive histogram (CLAHE) equalization. Smooth regions become noisier with adaptive histogram equalization. CLAHE may enhance noise in hectic circumstances. Histogram size may be limited by CLAHE. Understand that deep learning variation is a major issue. Use two tag techniques for variety. Match the center to the background to reduce variation. This study employed the dice coefficient loss function used by picture segmentation pros. The experiment suggests labeling may be better than initial marking in cases with insufficient data. Medical images are hard to classify and find. Everyone agrees transferring less data is hard. Semi-supervised learning overcomes auto-labeling naming issues. Proposed study successfully locates the lung using ROI segmentation from CT scans. Process attention model. The ROI segmentation model during data processing may find lung tumors, study suggests.

## 4 Model parameters and discussions

### 4.1 LUNA-16 dataset

A total of 5,000 CT scans were obtained from LUNA-16. Four expert radiologists annotated the images in the LIDC/IDRI database for 2 years (20–22). Each radiologist diagnosed the nodules as non-nodules, nodules with a diameter of ≤ 3 mm, or nodules with a diameter of ≥3 mm (23). This article examines the annotation process in detail. Three of every four nodules larger than 3 mm in diameter must be identified by radiologists (24). Non-standard findings have not been noted before (non-nodules, nodules < 3 mm, and nodules annotated by only one or two radiologists). Table 2 shows various illustrations of nodules in the LUNA-16 dataset.

**Table 2:** Various benigna and malignant nodules present in the LUNA-16 dataset.

**S. No**.	**Nodule name**	**Nodule image**
1	Small nodule	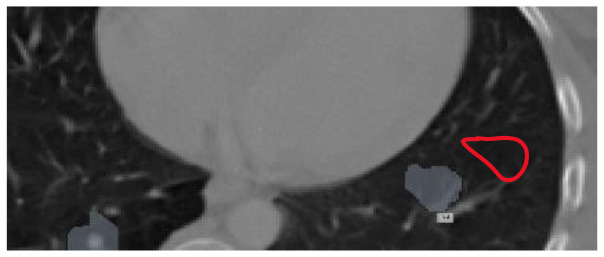
2	Ground glass opacity nodule	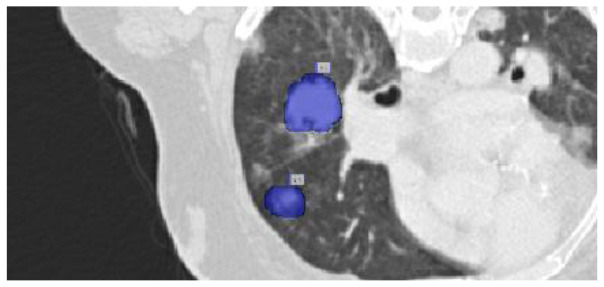
3	Rough edged nodule	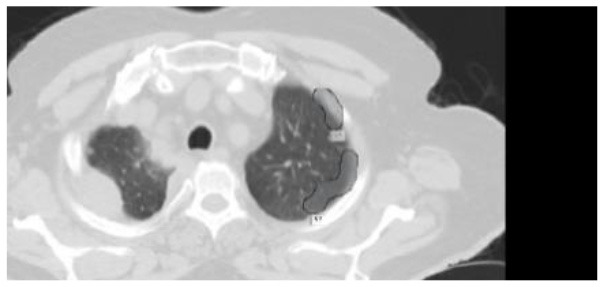
4	Thick walled nodule	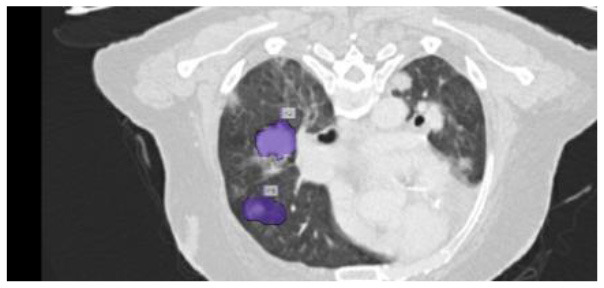
5	Granular nodule	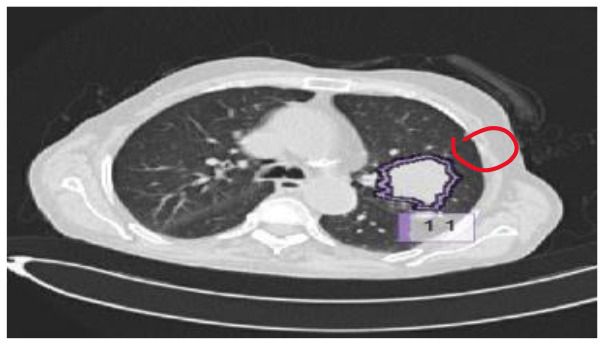
6	Pleural surface nodule	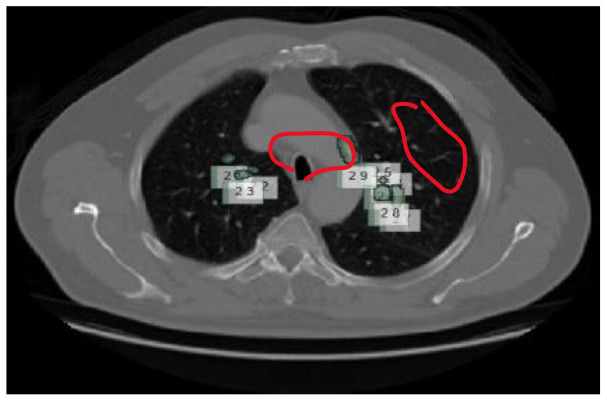
7	Pulmanory region nodule	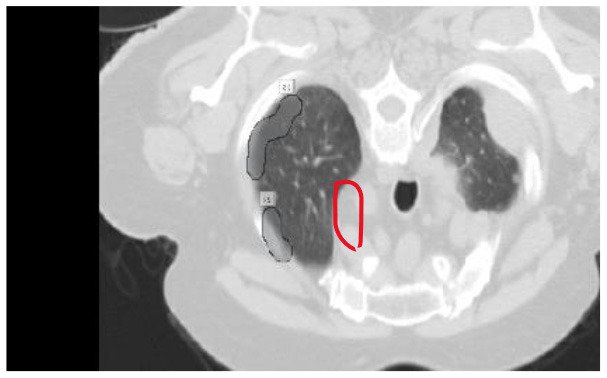

Table 3 presents various feature extraction values obtained from the LUNA16 database. A node, which refers to a specific structure, has a wide range of characteristics, with malignancy being used as an example to illustrate this. The estimation of the node's outline coordinates is utilized, whereas the surrounding area of the nodule is often underestimated. Lobulation refers to the configuration and attributes of a nodule. The measurement of a nodule in millimeters determines its diameter, which in turn determines its length. The border of the nodule indicates a transparent region.

**Table 3:** Presents the standard deviation of various features in LUNA-16/LIDC-IDRI dataset.

**Features in LUNA-16**	**Testing**	**Training**
Malevolence	1.98 ± 0.95	1.65 ± 1.03
Conjecture	2.60 ± 0.70	2.65 ± 0.77
Subtlety	1.89 ± 0.74	3.65 ± 0.69
Lobulation	2.73 ± 0.67	2.36 ± 0.71
Diameter in mm	9.17 ± 3.51	8.56 ± 0.56
Margin	3.03 ± 1.56	3.68 ± 0.58

Table 4 describes the dataset used in our study. We compiled a custom LUNA-16 dataset by combining annotated lung CT scans from various sources, including LIDC-IDRI datasets. This dataset comprises 5,000 annotated CT scans slices, each with a resolution of 512 × 512 pixels. The images were annotated by expert radiologists using semi-automated tools, ensuring high quality labels for training and evaluation.

**Table 4:** Dataset details.

**Dataset name**	**Description**
LUNA-16	Comprising annotated lung CT scans collected from partnering medical institutions, Includes data from LUNA 16 and LIDC-IDRI
Number of samples	5,000 annotated CT scans slices
Image resolution	128 × 128
Annotation methods	Expert radiologists using semi-automated tools
Preprocessing steps	- Slice normalization - Rescaling to uniform dimensions - Augmentation for training set

### 4.2 Study design

**Figure 4:**
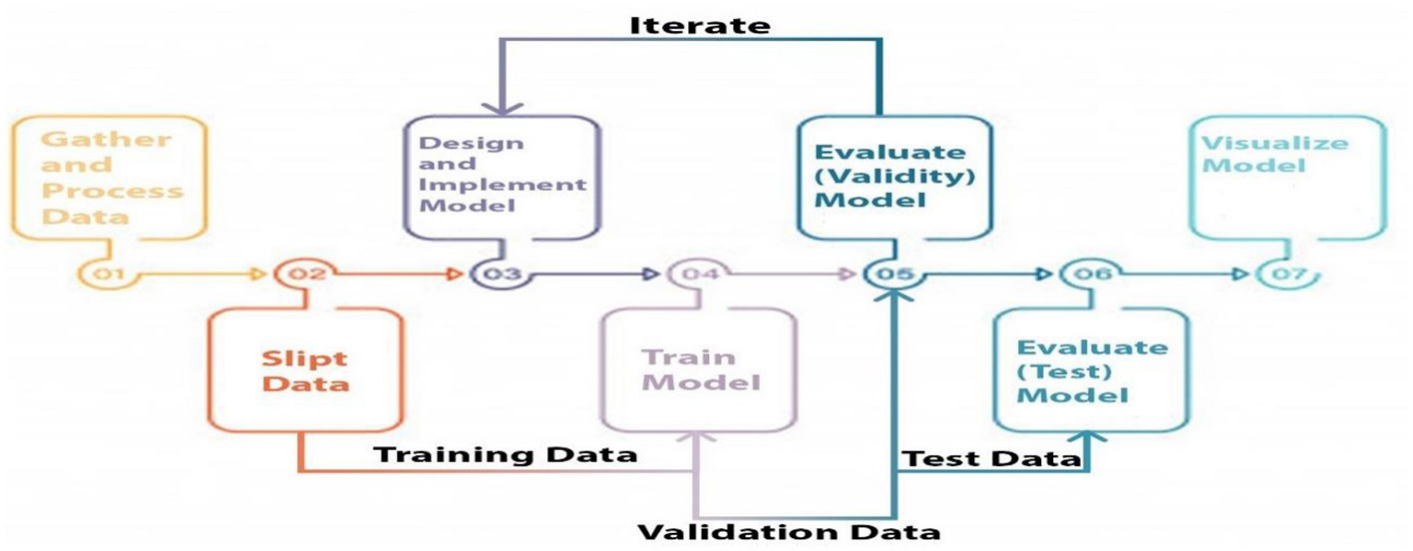
Proposed model study design and training, testing and validation process.

Three categories of data were created, namely training, validation, and testing. We built a model, trained it using validation data, and tested it. This method is repeated until a firm understands how our model reacts in real-world scenarios. Allow average pooling and expand the size of the final output by using layers in the filter. We examined our test data to determine what we could learn from it in order to enhance the model. Because we are neither testing nor training a model on a test dataset, we can utilize it only once per session. Two-parameter and three-parameter mixtures generate a model using a single test dataset, which significantly reduces the time and effort required. Figure 4 illustrates the study design.

### 4.3 Split and pre-process data

Jpeg serves as the data transport format in our architecture in the same way as DICOM. The Neuroimaging Informatics Technology Initiative (NIFIT) (25) is a 501(c)(3) not-for-profit organization committed to the advancement of neuroimaging informatics (NITI) (26). Despite its origins in neuroimaging, it is now commonly used in brain and other medical imaging. By memorizing the coordinates, it is possible to relate pixel values (i, j, k) to the position space (x, y, z) (x, y, z). Each data scan may provide three-dimensional medical images comprising 128 × 128 slices of varying thicknesses. Additional RAM is required to store the data in the DICOM format.

CLAHE12 contribute to the enhancement of CT scan quality (Contrast Limited Adaptive Histogram Equalization). The artwork places a premium on contrast and visual detail. The Hounsfield center values for the lung window and soft tissue were 600, 1,500, and 50,400. As a result, the lung window is the most frequently used Hounsfield range for lung image diagnosis. As shown in Table 1, the Hounsfield values of various body components were dispersed. Following sampling, the objective was to compress a snapshot to preserve the memory. Standardization is the next step in reducing computing costs. Subsequently, CLAHE was used to enhance nodule contrast and visibility.

**Figure 5:**
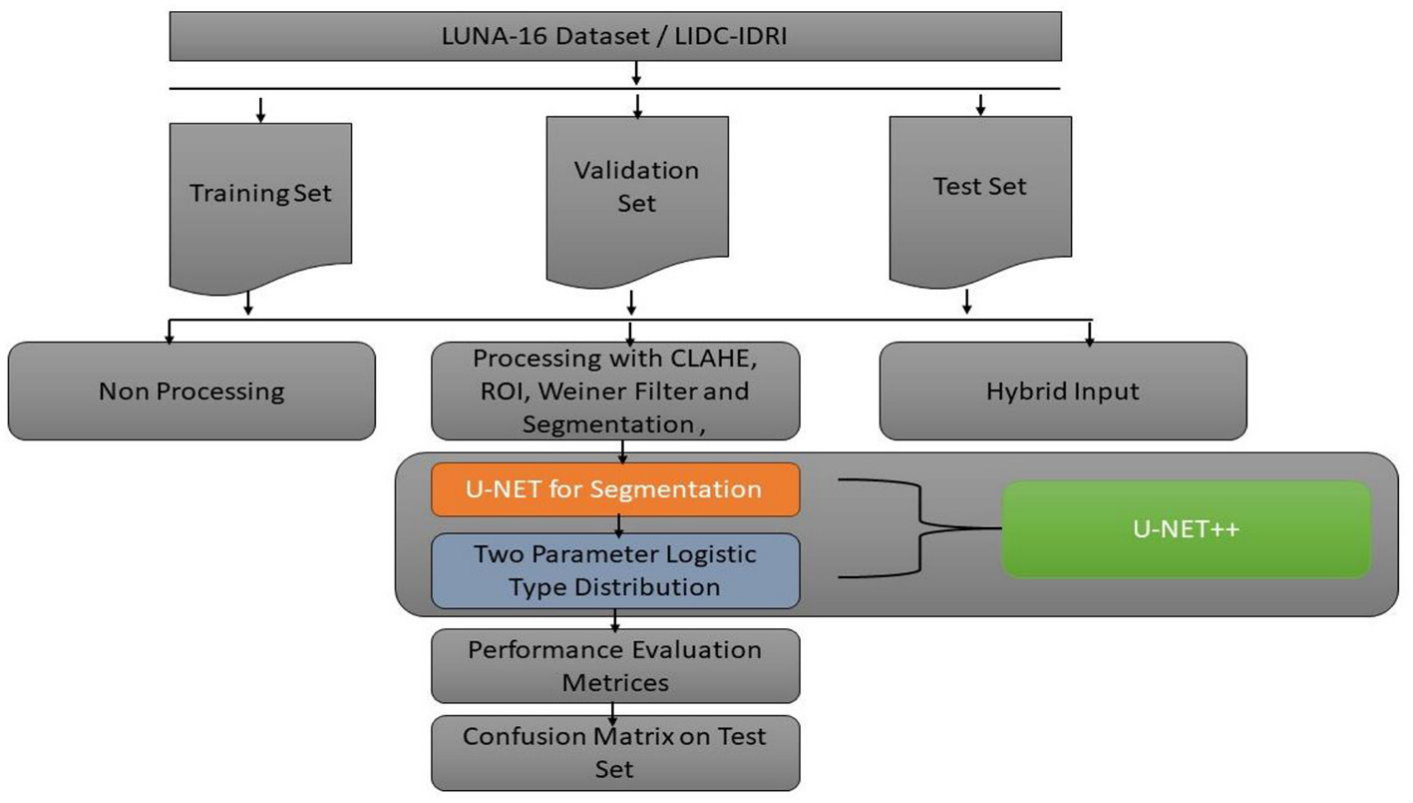
Flowchart and pre-processing steps.

Contrast-limited adaptive histogram equalization (CLAHE) has been used in image processing for a long time. Instead of adaptive Histogram Equalization (AHE13) (27), it cannot be used. Standard adaptive equalization may amplify noise in ordinarily homogeneous areas of the image. Consequently, the histogram tends to focus on this region. The CLAHE has the potential to enhance noise in locations where it is almost continuous. In Figure 5, the LUNA-16 dataset is preprocessed using the Wiener filter and CLAHE.

**Figure 6:**
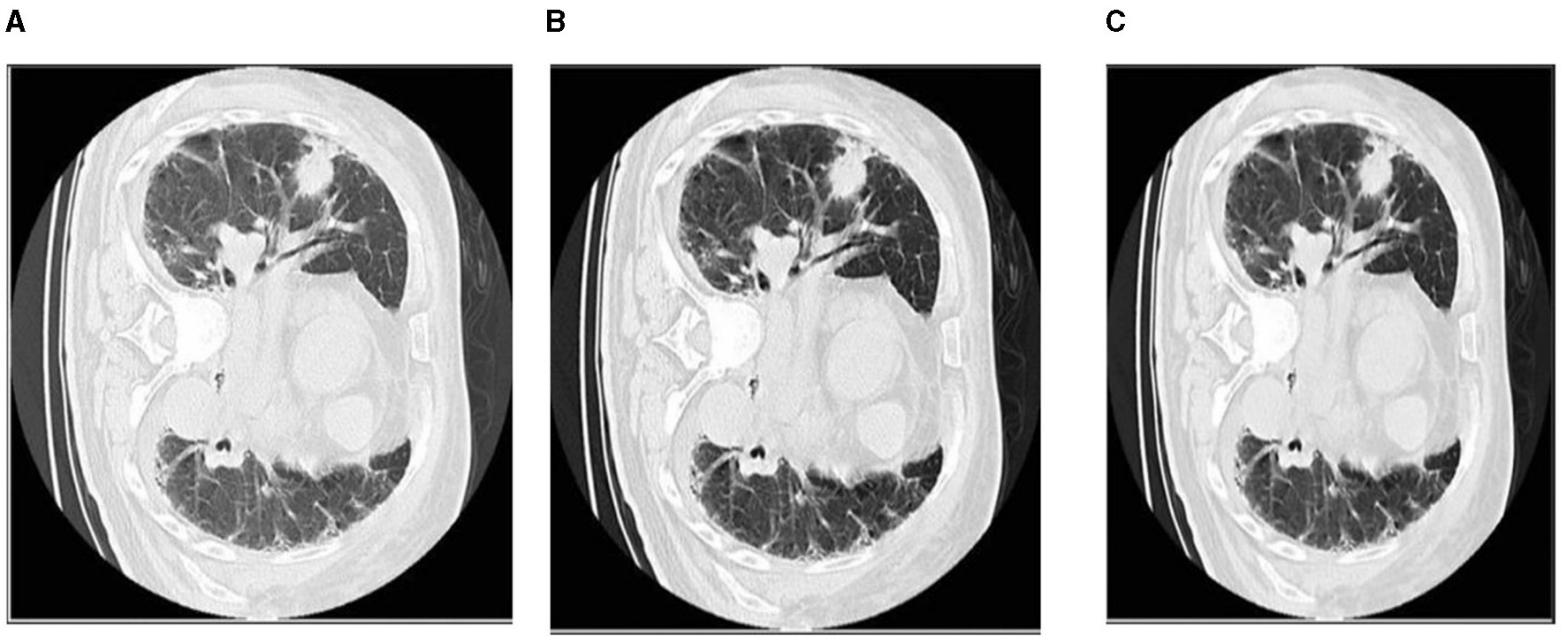
The first picture from left to right shows how the Wiener filter works with CLAHE. **(A)** Original CT scan image. **(B)** CT image with CLAHE image. **(C)** CLAHE with Weiner filter.

The CLAHE approach can be used to decrease the histogram concentration. When utilizing CLAHE, the concentrated histogram component was maintained. On the other hand, the exceeding histogram was maintained and equally distributed throughout all histogram bins. The Wiener filter is an extremely successful technique for visual noise reduction. PET/CT scans were afflicted with an additive noise of constant intensity. Figure 6 shows an example of the original CT scan image, second image is with CLAHE and third one is with CLAHE and weiner.

### 4.4 Architecture and implementation

The lung segmentation method utilized in this study used 5,000 lung CT scan images and masks. Each CT scan image has a resolution of 128 × 128 pixels. Images s black and white the final consequence is a split lung. The technique begins with the data being saved in memory and each image being resized to 32 × 32 pixels. Image processing was accelerated by shrinking the photographs. The images were corrected after rescaling. Subsequently, the dataset was partitioned into 70 percent training set and 30 percent test set. Rotation was performed to increase the number of training samples. There were eight rotating copies for each training sample. In Table 5, U-NET++ is composed of layer blocks that compress and stretch clockwise. The augmented dataset was initially used to define the input layer. The following are the layers of convolution, non-linearity, and down sampling. Non-linearity is first applied to decrease the final image size, followed by convolution to apply a filter, and finally max-pooling. The image is concatenated by applying similar layers in contracting and expanding patterns, and then up-sampled to make it larger. The output layer provides a lung segmentation image. After all layers have been trained, the U-Net ConvNet is created (28). For example, using Adam as the optimizer, the dropout was set to 0.5, epochs were set to 10, and steps per epoch were set to 200 (29). Each layer, similar to the model architecture, has its own set of filters. We examined the performance of U-Net ConvNet using test data.

**Table 5:** Proposed network architecture with two parameters distribution.

**Layer**	**Type**	**Input size**	**Output size**	**Kernal size**	**Stride**	**Padding**
**U-NET**++ **down sampling encoder process**						
Layer 1	Conv+ReLU	128 × 128	128 × 128	3 × 3	1	1
Layer 2	Conv+ReLU	128 × 128	128 × 128	3 × 3	1	1
Layer 3	Max Pooling	128 × 128	64 × 64	2 × 2	2	0
Layer 4	Conv+ReLU	128 × 128	64 × 64	3 × 3	1	1
Layer 5	Conv+ReLU	64 × 64	64 × 64	3 × 3	1	1
Layer 6	Max Pooling	64 × 64	32 × 32	2 × 2	2	0
Layer 7	Conv+ReLU	32 × 32	32 × 32	3 × 3	1	1
Layer 8	Conv+ReLU	32 × 32	32 × 32	3 × 3	1	1
Layer 9	Max Pooling	32 × 32	16 × 16	2 × 2	2	0
Layer 10	Conv+ReLU	16 × 16	16 × 16	3 × 3	1	1
Layer 11	Conv+ReLU	16 × 16	16 × 16	3 × 3	1	1
Layer 12	Max Pooling	16 × 16	8 × 8	2 × 2	2	0
Layer 13	Conv+ReLU	8 × 8	8 × 8	3 × 3	1	1
Layer 14	Conv+ReLU	8 × 8	8 × 8	3 × 3	1	1
Layer 15	Max Pooling	8 × 8	4 × 4	2 × 2	2	0
**U-Net**++ **up-sampling decoder process**						
Layer 16	Up sample Transposed Conv	4 × 4	8 × 8	2 × 2	2	0
Layer 17	Conv+ReLU	8 × 8	8 × 8	3 × 3	1	1
Layer 18	Conv+ReLU	8 × 8	8 × 8	3 × 3	1	1
Layer 19	Up sample Transposed Conv	8 × 8	16 × 16	2 × 2	2	0
Layer 20	Conv+ReLU	16 × 16	16 × 16	3 × 3	1	1
Layer 21	Conv+ReLU	16 × 16	16 × 16	3 × 3	1	1
Layer 22	Up sample Transposed Conv	16 × 16	32 × 32	2 × 2	2	0
Layer 23	Conv+ReLU	32 × 32	32 × 32	3 × 3	1	1
Layer 24	Conv+ReLU	32 × 32	32 × 32	3 × 3	1	1
SoftMax function	Convolutional Layer_8	32 × 32	32 × 32	3 × 3	1	1
Benign or malignant	SoftMax Function	32 × 32	32 × 32	3 × 3	1	0

There were five columns in total. The first column provides the layer name, followed by the number of filters, filter type/size, dimension, and concatenated layers. Eleven convolutional layers were used. The input layer is the first layer. A 32 × 32-pixel input layer is displayed in this picture. For the Con1 layer, eight 3 × 3 filters are needed. The size of the images remained unchanged. Con1 was closely related to other con1. After the con layers, there were ReLU layers.

#### 4.4.1 Simulation settings

To facilitate the replication of our work, we provide a detailed description of the simulation settings and the dataset used. This information includes hardware and software configurations, data preprocessing steps, and hyperparameter settings.

The simulation settings outlined in Table 6 provides comprehensive details on the hardware software environment used for our requirements. Our setup included an Intel core i9-10900k CPPU and an NVIDIA GEFORCE RTX 3090 GPU, ensuring sufficient computational power for training deep learning models. We utilized Ubuntu 20.04 LTS as our operating system, with python 3.8 and TensorFlow 2.4 for model development and training.

**Table 6:** Simulation setting used in our proposed work.

**Component**	**Description**
**Hardware**	
CPU	Intel Core i9-10900K
GPU	NVIDIA GeForce RTX 3090
RAM	64 GB DDR4
**Software**	
Operating System (OS)	Ubuntu 20.04 LTS
Programming language	Python 3.8
Deep learning framework	TensorFlow 2.4, Keras
**Data preprocessing**	
Normalization	Rescale pixel values to range [0,1]
Augmentation techniques	Rotation, translation, flipping, scaling
Data split	70% training, 15% validation, 15% testing
**Model training**	
Optimizer	Adam
Learning	0.001
Batch size	32
Epochs	100
Loss function	Dice loss
Metrics	Dice co-efficient, IoU, Sensitivity, Specificity

Table 7 details the hyperparameters and model configuration. We implemented a U-NET++ with 20 layers, utilizing a kernal size of 3 × 3 and max pooling layer of 2 × 2. The ReLU activation function was used throughout the network, with a sigmoid activation function in the output layers for binary segmentation (30). A dropout rate of 0.5 and L2 regularization were applied to prevent overfitting.

**Table 7:** Hyperparameters and model configurations.

**Parameter**	**Values**
Network architecture	U-NET++
Number of layers	20
Kernal size	3 × 3
Pooling	Max pooling (2 × 2)
Activation function	ReLU (Rectified Linear Unit)
Output layer activation	Sigmoid
Dropout rate	0.5
Regularization	L2 regularization with delta = 0.001

These settings and configurations provide a robust framework for replicating our lung cancer segmentation model and can serve as a foundation for further research and development in this domain.

### 4.5 Training process

**Figure 7:**
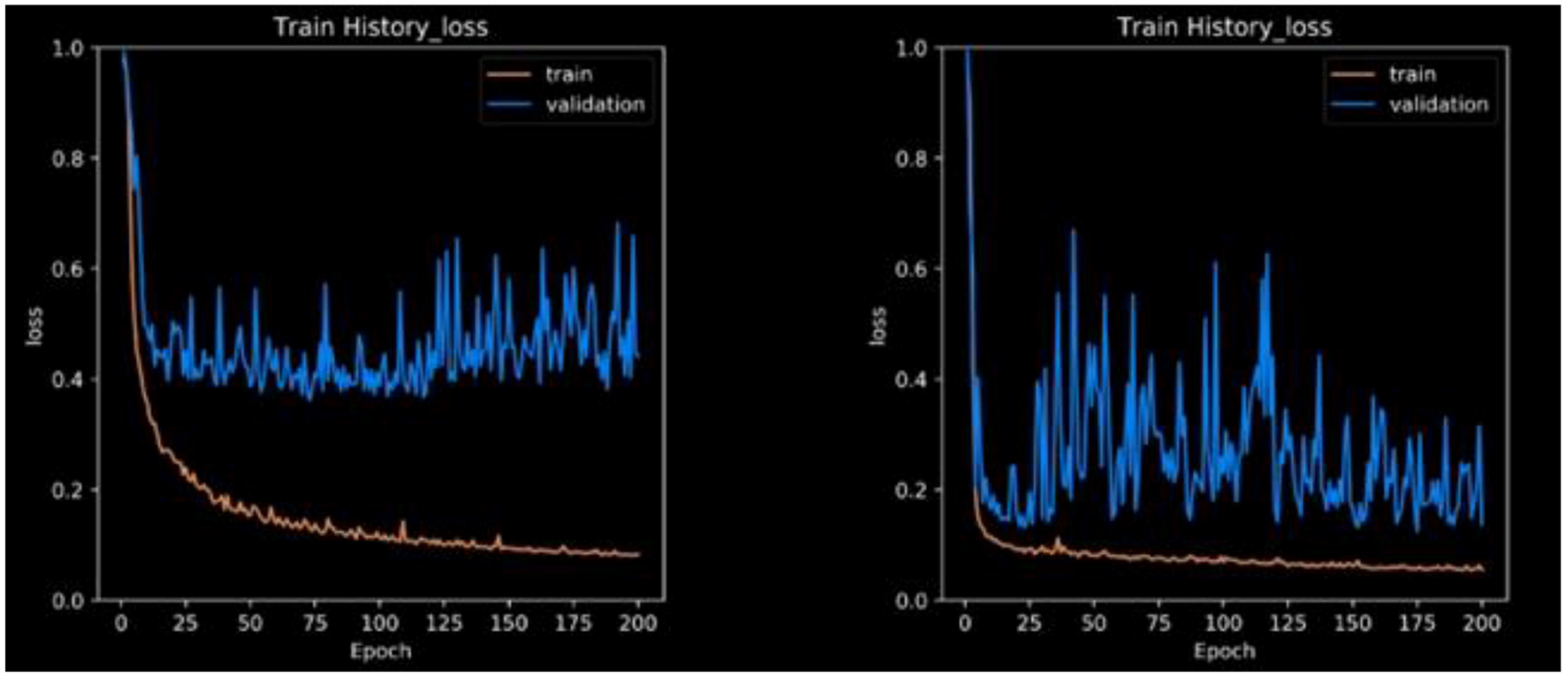
The proposed framework with respect to both training and validation accuracy.

The loss function expresses the loss of the die coefficients. Frequently, the dice coefficient is used to segment medical images, as shown in Figure 7. It is often used to compare two samples. This experiment generated sufficient compelling evidence to be deemed to be conclusive.

This research is mostly concerned with two-dimensional pictures. It might end up saving a lot of money in the long term. Another example is graphics processing unit (GPU) throttling. Owing to memory limitations, the majority of GPUs have difficulty in training 3D models. 2D and 3D models are available for downloading in various formats. We break down our findings into different segmentation strategies with an emphasis on unbalanced and tiny datasets. In addition, the model training process converged in 200 epochs. The confusion matrix can be used to evaluate real-world data and calculate metrics such as accuracy, sensitivity, and specificity. The testing loss is approximately 0.4 in Figure 6, whereas CLAHE and Wiener may be as low as 0.1 without pre-processing.

## 5 Results discussion and comparison with other models

**Figure 8:**
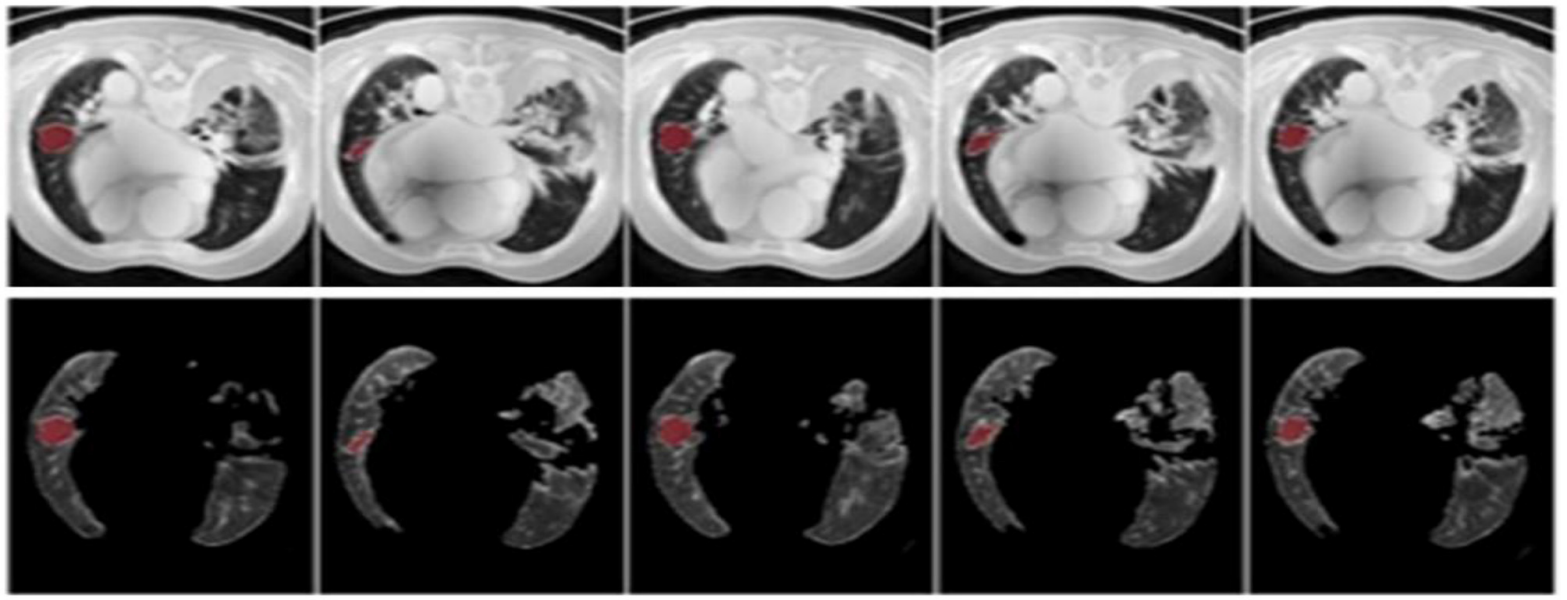
Prior to and during the segmentation procedure, the ground-truth forecast was used in each of these instances.

The results were enhanced by using the ROI segmentation method. It seems that it has the capacity to address the problem of the model's inaccurate positioning of labels. As a consequence, following the recommended methodology may lead to decreased losses. Furthermore, it was shown that the training session continued to slow down. The lesson is enhanced in its effectiveness as shown in Figure 8. It is advisable to apply the same treatment to both one-dimensional and two-dimensional data. The objective of this strategy is to eliminate any errors in labeling in both directions. Over time, there was a gradual reduction in the size of each point. Engaging in conversations with individuals helps achieve both objectives.

If the dataset is insufficient, it may be necessary to round up more labels. Overall, there were 159 cancerous tumors, and the standard deviation of the Dice coefficient was 0.2. Although its model had a low mFPI, the DL-based model was successful in detecting lung tumors from chest X-rays, the results are shown in Figure 8. The evaluations of the proposed models are presented in Table 8.

**Table 8:** The evaluation report of the different lung nodule semantic segmentation with comparison to our proposed algorithm.

**Evaluation**	**Cai et al. (10)**	**Banu et al. (11)**	**Proposed model**
Dice similarity index	87.22%	90.24%	91.76%
Error matrix	Accuracy 90%	Accuracy 89%	Accuracy: 90%
	Sensitivity 90%	Sensitivity 90%	Sensitivity: 89%
	Specificity 89%	Specificity 86%	Specificity: 90%

TensorFlow was used to evaluate the effectiveness of the U-NET++ approach for the segmentation of lung tumors. The evaluation was performed with the assistance of an image segmentation examiner. Images from LUNA-16 were used to complete the segmentation process. The results of the logistic distributions with the two parameters are shown in the following table. Based on the information shown in Table 9, it is presumed that the intensities of the image pixels adhere to a combination of logistic-type distributions with two parameters.

**Table 9:** The refined value of k with two-parameter U-NET architecture.

**Constraints**	**First parameters**		**Revised calculations**									
	**Image region**		**Image area**									
	**1**	**2**	**1**	**2**	**1**	**2**	**1**	**2**	**1**	**2**	**1**	**2**
α_*i*_	0.500	0.500	0.2588	0.7428	0.500	0.500	0.2588	0.7428	0.500	0.500	0.2588	0.7428
μ_*i*_	60.54	121.98	19.48	136.18	60.54	121.98	19.48	136.18	60.54	121.98	19.48	136.18
$\sigma_{i}^{2}$	94.2568	128.784	124.281	117.251	94.2568	128.784	124.281	117.251	94.2568	128.784	124.281	117.251

**Figure 9:**
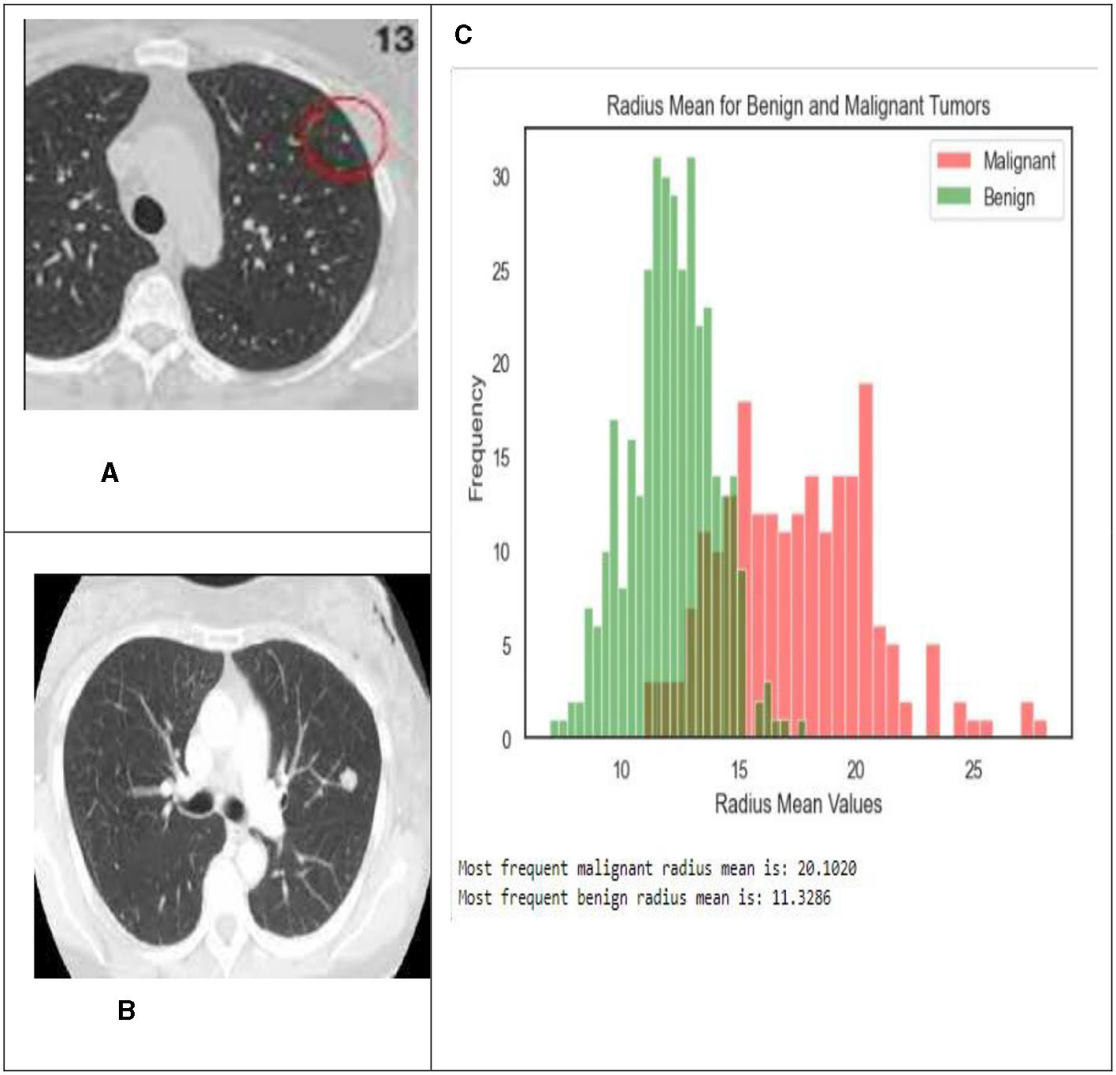
In this illustration, the pixel intensities generated from CT scan images of lung nodules that were either benign or malignant were included. **(A)** Malignant tumor. **(B)** Benign tumor. **(C)** Shows the radius mean for benign and malignant tumors.

The pixel intensities in each of the k sectors of the image were assumed to follow a two-parameter logistic distribution, with unique parameters. This assumption was based on the fact that a picture. The histogram of pixel intensities was analyzed to estimate the segment count for each CT scan image used in the experiment. The histograms that indicate the pixel intensities that may be observed in the CT scan images are shown in Figure 9.

Typically, malignant tumors have higher average radius values compared to benign tumors, as seen by histograms and bar graphs. The average radius of malignant tumors is 20.1020, whereas benign tumors normally have a radius of 11.3286. These data indicate the differences in average radius values between benign and malignant tumor types.

### 5.1 Visualization of the model

After examining the data, they found a connection, as shown in Table 10, between how well the suggested method worked and other ways of showing the same thing. To determine how well the U-NET++ model segmented the LUNA16 trial dataset, five radiotherapists were used for comparison with real experts. Of the three radiologists, 81.26% were good at segmenting patients. The U-NET++ model was also tested by comparing it with the U-NET model and many other benchmark models, such as the newest ResNet152V2.

**Table 10:** Comparing the proposed model's quantitative segmentation results to well-established benchmark models.

**References**	**Classifier models**	**Dice coefficient (%)**	**Sensitivity (%)**	**Specificity (%)**
Huang and Hu (6)	NU-NET	89.26 ± 12.45	89.63 ± 23.56	89.21 ± 14.25
Zhao et al. (7)	U-NET	76.24 ± 17.89	85.45 ± 12.54	88.24 ± 15.45
Chiu et al. (8)	2D U-NET	81.89 ± 14.56	91.25 ± 12.89	78.26 ± 15.45
Gao et al. (9)	U-NET	86.45 ± 56.78	78.56 ± 23.57	87.65 ± 23.90
Cai et al. (10)	U-NET	87.22 ± 56.45	75.67 ± 23.74	56.24 ± 22.56
Banu et al. (11)	3D U-NET	90.24 ± 24.45	80.26 ± 23.77	79.23 ± 22.74
Xia (12)	WU-NET	83.12 ± 25.06	88.96 ± 26.32	80.24 ± 23.56
***Proposed work***	***U-NET++***	***91.76** **±26.67***	***89.54** **±3.65***	***85.98** **±25.98***

Bold values indicate highest compared with other classifiers.

**Figure 10:**
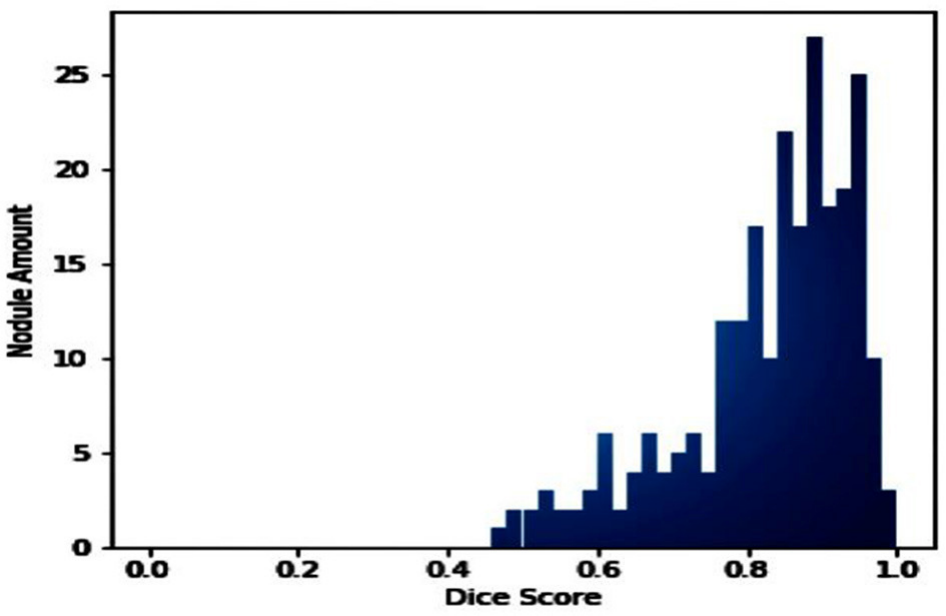
The frequency of lung CT scans was examined in the LUNA16 collection.

The number of nodes, Dice coefficient value index, and distribution are presented in Figure 10. This allowed the U-NET++ model to be tested on a test set. Giving each node a number and placing it in the midst of a test set trial is standard.

Duan et al. (23) employed a U-NET architecture with advanced deep learning techniques, resulting in a dice co-efficient of 0.88. Similarly, Duan et al. (23) utilized V-NET incorporating 3D convolutional layers, achieving a dice co-efficient of 0.90. The method by Petit et al. (25) leveraged transformer networks, while Ali et al. (26) utilized efficient net for a more parameter efficient approach.

Table 11 shows a numeric comparison of how well the new method U-NET++ works with three other deep learning models, U-Net (7), NU-Net (6), and WU-Net (12), using CT images of lung nodules from a dataset that was already made public, the suggested method is better than the average method for segmenting images of lung nodules.

**Table 11:** Quantitative evaluation of lung cancer segmentation methods based on key performance metrics, model architectures and unique features.

**Method**		**Score**			**96% C.I for mean**	
		**Mean**	**St. deviation**	**St. error**	**Lower bound**	**Upper bound**
IoU	**Proposed methods**	**0.879**	**0.105**	**0.049**	**0.748**	**0.925**
	U-Net (7)	0.805	0.104	0.073	0.677	0.916
	NU-NET (6)	0.780	0.117	0.062	0.680	0.824
	WU-NET (12)	0.731	0.144	0.088	0.613	0.865
Recall	**Proposed method**	**0.933**	**0.035**	**0.043**	**0.814**	**0.963**
	U-Net (7)	0.870	0.022	0.038	0.759	0.955
	NU-NET (6)	0.850	0.027	0.030	0.755	0.967
	WU-NET (12)	0.805	0.154	0.83	0.702	0.954
Precision	**Proposed method**	**0.950**	**0.130**	**0.040**	**0.834**	**0.991**
	U-Net (7)	0.890	0.106	0.029	0.780	0.992
	NU-NET (6)	0.880	0.086	0.024	0.770	0.996
	WU-NET (12)	0.831	0.154	0.062	0.704	0.946
F1-Score	**Proposed method**	**0.940**	**0.120**	**0.040**	**0.826**	**0.993**
	U-Net (7)	0.886	0.086	0.023	0.754	0.975
	NU-NET (6)	0.851	0.117	0.035	0.789	0.923
	WU-NET (12)	0.813	0.128	0.057	0.721	0.948

Bold values indicates is highest compared with other classifiers.

We used Fisher's least significant difference (LSD) method in SPSS software to look at the numeric results and see if the suggested way in Table 12 worked. By using the LSD test, we can see that the suggested method does better than standard methods in terms of IoU, recall, precision, and F1-score (*p* < 0.001).

**Table 12:** Statistical analysis.

**Multiple comparisons**					**96% C.I for mean**	
**Dependent**	**Model(a)**	**Methods(b)**	**Mean difference**	**Sig**	**Lower bound**	**Upper bound**
IoU	Proposed model	U-Net (7)	0.072^#^	< =0.001	0.061	0.094
		NU-NET (6)	0.096^#^	< =0.001	0.084	0.125
		WU-NET (12)	0.145^#^	< =0.001	0.125	0.165
Recall	Proposed model	U-Net (7)	0.055^#^	< =0.001	0.038	0.074
		NU-NET (6)	0.061^#^	< =0.001	0.050	0.075
		WU-NET (12)	0.115^#^	< =0.001	0.103	0.135
Precision	Proposed model	U-Net (7)	0.056^#^	< =0.001	0.045	0.078
		NU-NET (6)	0.065^#^	< =0.001	0.055	0.085
		WU-NET (12)	0.122^#^	< =0.001	0.104	0.145
F1-Score	Proposed model	U-Net (7)	0.048^#^	< =0.001	0.038	0.065
		NU-NET (6)	0.072^#^	< =0.001	0.065	0.089
		WU-NET (12)	0.121^#^	< =0.001	0.112	0.137

^#^Indicates proposed Method is better than the existing classifiers.

**Figure 11:**
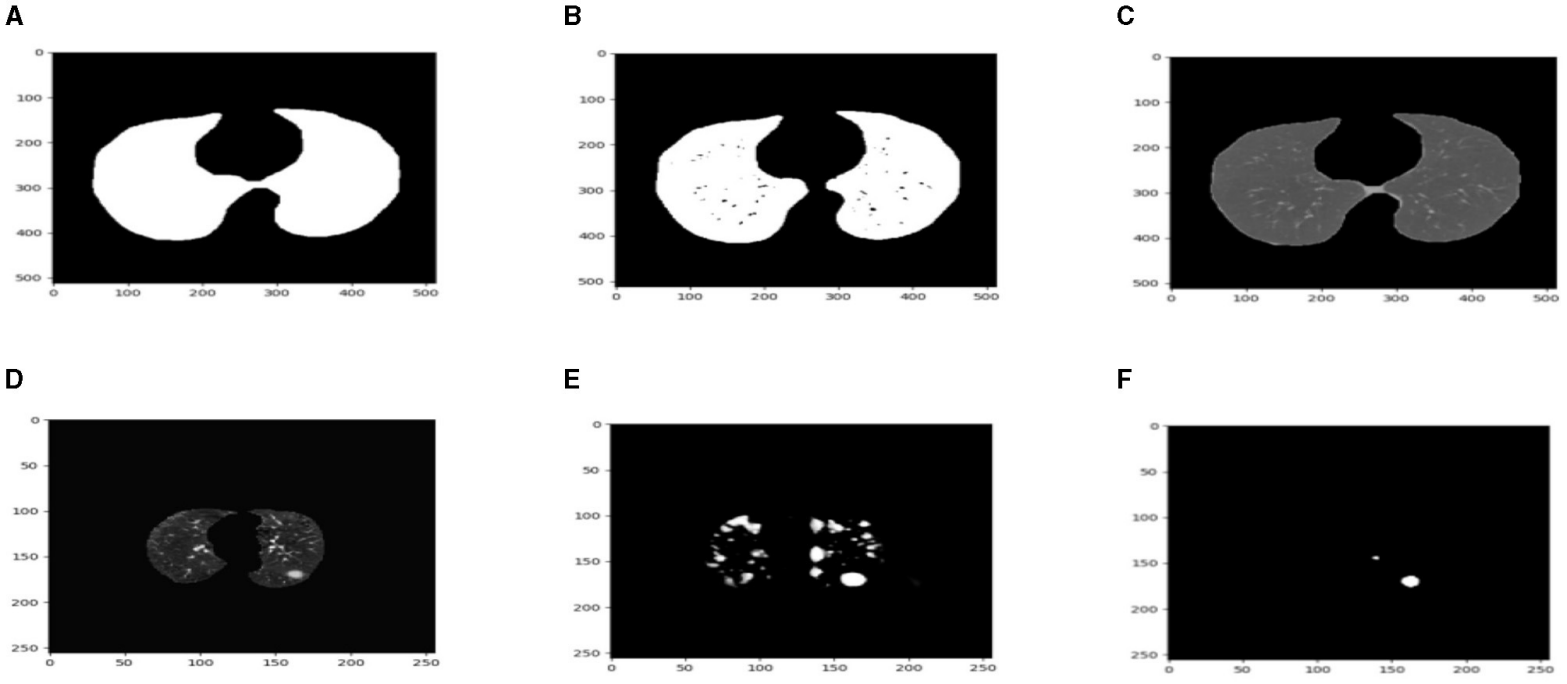
Utilizing the provided approach, we performed visual segmentation of heterogeneous lung nodules. **(A)** Clustered image. **(B)** Segmented image. **(C)** Extracted image. **(D)** Extracted image with nodules localizations. **(E)** Nodule capture. **(F)** Nodule region highlighted.

After preprocessing the image, shown in the Figure 11A the grouped picture, Figure 11B what was found when Lung tumors were identified. Figure 11C results of cutting lung tumors into whole pieces. Figure 11D the findings of the lung tumor search. Figure 11E picture showing the effects on a specific area of lung tumors when they are cut into pieces. Figure 11F a picture of a lung tumor that was accurately cut into pieces.

Our Model, built on a U-NET++ architecture, demonstrated a baseline performance with a dice-coefficient of 91.76% and an IoU of 89.78%. Recent methods, such as the swin Transformer by Ronneberger et al. (27), achieved higher performance metrics through the use of advanced architectures and techniques.

**Figure 12:**
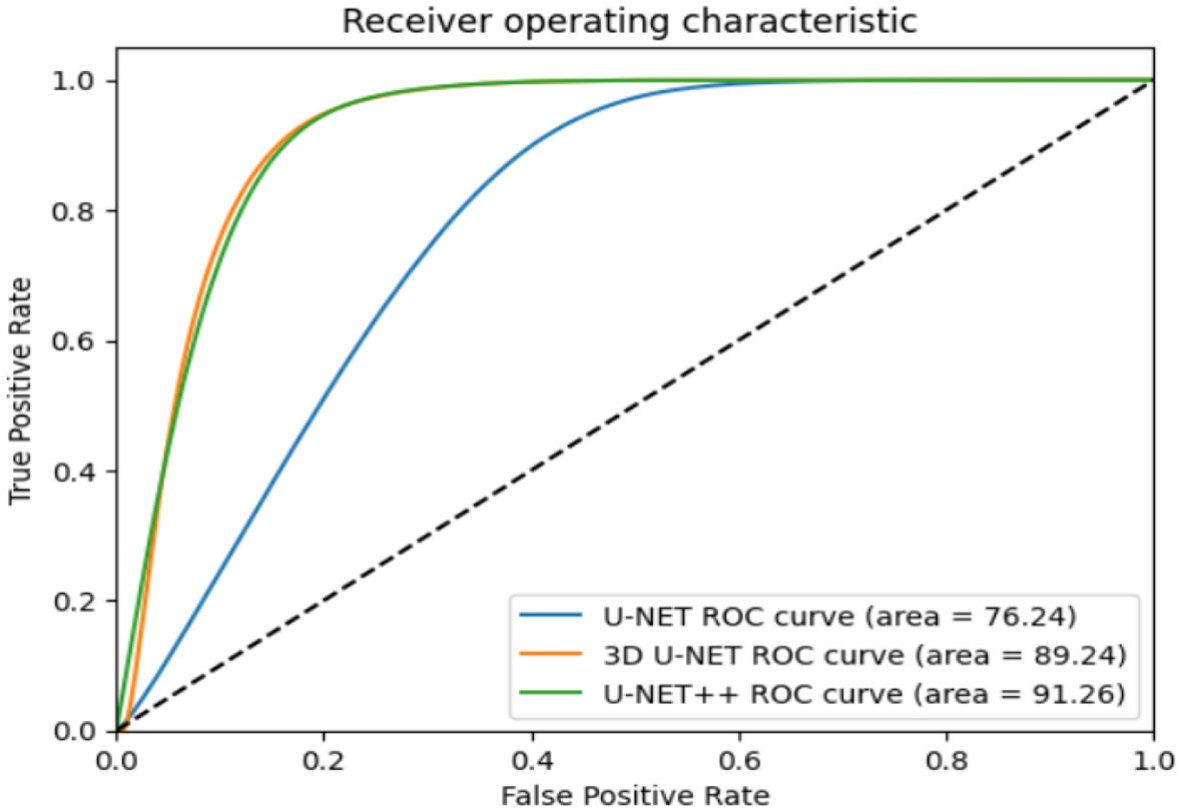
AUC curve for the proposed classifier with respective to other classifiers.

The images in Figure 12 show a DSC value of at least 0.8 can be trusted for most tumors. The dice index results were compared with the U-NET++ architecture's specific performance to ensure that the model's results were correct. The Dice similarity score (DSC) for the U-NET++ model was 90.84%, which is an unusually high level of success. Because it has fewer parameters than the original U-NET design, the U-NET++ model can effectively separate features and divide them into groups.

The ROC curves in Figure 11 demonstrate that radiologists have the capacity to obtain much greater levels of specificity (i.e., decreased false positive rates) with a low impact on sensitivity (31). By narrowing down the requirement for a positive screen for individuals who are recommended to undergo repeat computed tomography (CT) scans, it is possible to achieve a specificity of 92.4%, while slightly decreasing the sensitivity to 86.9%.

## 6 Conclusions and future work

Lung segmentation is necessary for the effective diagnosis and identification of lung disorders. There has been a frenzy of lung segmentation research over the past few years, all aimed at improving the accuracy. To identify and categorize lung illnesses, automated analysis of a CT scan must first “segment” the lung. The precision at which lung segmentation can be performed has been the subject of several studies. Deep learning algorithms and basic thresholding approaches have been applied to lung segmentation. U-NET++ is particularly effective in separating cells and neurons from images acquired using a PET Scan. In this study, U-NET++ was used for lung segmentation. The accuracy of the lung segmentation using U-NET++ was 91%. The original purpose of U-NET++ was to separate tiny images. The lungs were effectively divided using CT images. By shrinking the images, they were reduced from 128 × 128 to 32 × 32 pixels. There were 25 convolutional layers in total in this network. It is much more accurate to train U-NET++ using an original image size of 128 × 128. The convolutional layers may be increased in size to enhance the accuracy of the filter.

## Data availability statement

The dataset used for the findings will be shared by the corresponding authors upon reasonable request.

## Author contributions

EA: Investigation, Software, Writing – original draft, Writing – review & editing. EN: Methodology, Software, Supervision, Writing – original draft, Writing – review & editing. NJ: Formal analysis, Project administration, Supervision, Writing – original draft, Writing – review & editing. SB: Project administration, Supervision, Validation, Writing – original draft, Writing – review & editing. HS: Resources, Validation, Writing – original draft, Writing – review & editing. SA: Conceptualization, Methodology, Visualization, Writing – original draft, Writing – review & editing. JN: Resources, Validation, Visualization, Writing – original draft, Writing – review & editing.
